# String Stability Analysis and Design Guidelines for PD Controllers in Adaptive Cruise Control Systems

**DOI:** 10.3390/s25113518

**Published:** 2025-06-03

**Authors:** Kangjun Lee, Chanhwa Lee

**Affiliations:** 1 Department of Artificial Intelligence and Robotics, Sejong University, Seoul 05006, Republic of Korea; kangjun.lee@sju.ac.kr; 2 Artificial Intelligence and Robotics Institute, Sejong University, Seoul 05006, Republic of Korea

**Keywords:** adaptive cruise control, string stability, individual vehicle stability, PD control, platoon

## Abstract

This paper proposes a practical design guideline for selecting control parameters in adaptive cruise control (ACC) systems to ensure both individual vehicle stability and string stability in vehicle following systems with homogeneous longitudinal dynamics. The primary control objective is to regulate spacing errors under a constant time-gap policy, which is commonly adopted in ACC applications. By employing a simple proportional-derivative (PD) controller, we present a clear methodology for tuning the proportional and derivative gains. The proposed approach demonstrates that string stability can be effectively achieved using this straightforward control structure, making it highly applicable for assisting practitioners in selecting appropriate parameters for real-world platooning scenarios. We provide a rigorous analysis of the necessary and sufficient conditions for selecting PD gains, along with practical guidelines for implementation. The effectiveness of the design guideline is further validated through simulations conducted in realistic driving scenarios.

## 1. Introduction

### 1.1. Adaptive Cruise Control

Adaptive cruise control (ACC) systems are a fundamental component of advanced driver assistance systems (ADAS) and autonomous driving technologies [1,2,3,4]. ACC systems represent a significant advancement over conventional cruise control systems by automatically adjusting vehicle speed to maintain a safe distance from preceding vehicles. Relying solely on on-board sensors such as radar, LiDAR, or cameras, ACC systems measure the relative distance and velocity to the vehicle ahead, enabling autonomous decision-making without requiring vehicle-to-vehicle (V2V) communication or infrastructure support. In the absence of any preceding vehicle, the ACC vehicle maintains a preset cruising speed, mimicking traditional cruise control. However, when a slower or nearby vehicle is detected in its path, the system switches from speed control to spacing control and transitions into the vehicle-following mode, adjusting the input commands to maintain a desired headway distance. The control strategy of this vehicle-following mode serves the main focus of this study.

ACC technology provides several advantages, making it a practical and effective component of modern intelligent transportation systems. One of its primary benefits is enhanced safety. Well-calibrated ACC systems are capable of responding to traffic dynamics more rapidly and consistently than human drivers, thereby reducing the risk of collisions [5]. This advantage is especially important considering that human error has been identified as the primary cause of most traffic accidents and fatalities, as demonstrated by numerous studies [6,7,8]. By reducing human involvement in driving tasks, ACC systems can substantially mitigate such errors and contribute to improved road safety. In addition to safety improvements, ACC enhances fuel efficiency by promoting smoother acceleration and deceleration profiles [9]. When deployed in platoon formations with even shorter inter-vehicle spacing, further energy savings can be achieved through aerodynamic drag reduction. This is particularly beneficial in commercial freight transport, where truck platooning not only lowers fuel consumption but also reduces operational costs [10,11,12,13,14]. Moreover, the impact of ACC on traffic flow has been extensively investigated [15,16]. Studies indicate that properly designed ACC control strategies can improve traffic efficiency by maintaining consistent and short inter-vehicle distances. This helps suppress traffic oscillations and prevents the emergence of stop-and-go waves, which are often caused by the variability in human driving behavior. As a result, ACC contributes to reduced congestion and improved roadway throughput, especially in high-density traffic conditions.

### 1.2. Background and Related Work

While cooperative adaptive cruise control (CACC) systems have been proposed to enhance performance by V2V communication for shorter headway spacing, CACC is not yet a practical solution for widespread deployment due to technological and infrastructural limitations in most production vehicles. Another major challenge for CACC is the time delay introduced by wireless networks, which many studies aim to overcome [17,18,19,20,21]. Although CACC enables vehicles to maintain short headway distances, any sudden disconnection in the network during unexpected situations can pose significant safety risks. Consequently, ACC continues to serve as a foundational and self-contained solution for longitudinal vehicle control. In real-world environments, communication links can be unreliable or completely unavailable, necessitating fallback to sensor-based ACC operation.

Conventional ACC has primarily focused on flexibly and safely adjusting the distance to the preceding vehicle to enhance passenger ride comfort [22,23]. However, in scenarios where multiple vehicles rely entirely on driver assistance systems to form a platoon, ensuring the safety of the entire vehicle string becomes critical. In such cases, string stability emerges as a key performance metric [24,25,26,27,28]. However, reference [29] demonstrates that a platoon of eight commercially available vehicles equipped with ACC fails to maintain string stability, which highlights that commercial ACC systems are primarily designed to ensure safe following of the preceding vehicle, rather than to maintain stability across the entire vehicle platoon. Consequently, the control strategies implemented in these commercial systems lack considerations necessary to maintain string stability across multiple vehicles. To address these practical issues, we propose an ACC controller architecture that guarantees both individual vehicle stability and string stability. The proposed design adopts the simplest controller form based on proportional-derivative (PD) control structure, which facilitates straightforward implementation in commercial applications.

### 1.3. Contributions of the Paper

Extensive research has been conducted on ACC, with numerous studies employing advanced control techniques such as model predictive control (MPC) [30,31,32] and $H_{2}$/$H_{\infty }$ control [33] to improve performance and robustness, including reductions in fuel consumption. However, these sophisticated strategies remain impractical for widespread commercial deployment and generally lack rigorous guarantees for string stability. Furthermore, most commercial ACC systems continue to rely on traditional proportional-integral-derivative (PID) control due to its alignment with real-time computational constraints in practical deployments. Motivated by this, we adopt PD controllers to maintain practical simplicity while meeting modest computational requirements. Although prior studies have proposed theoretical conditions for achieving string stability with PD control [27,34], they do not provide explicit analytical bounds on the allowable ranges of the PD gains, leaving practical implementation and gain selection largely unresolved. Moreover, the relationship between control gain selection and string stability performance has not been systematically explored in a manner accessible to practitioners. This gap highlights the need for a more practical and transparent design methodology tailored to PD control architectures in ACC systems.

In this paper, we address this need by proposing a systematic design guideline for selecting the control gains of a PD-based ACC system operating under the constant time-gap (CTG) policy, which is a widely adopted strategy in real-world ACC implementations. We focus on vehicle strings with homogeneous longitudinal dynamics and develop a rigorous yet practical procedure for determining proportional and derivative gains that ensure both individual vehicle stability and string stability. Our analysis revisits and formalizes the necessary and sufficient conditions for string stability through frequency domain methods based on transfer function analysis. The resulting guideline is validated through simulations in realistic driving scenarios. The primary contribution of this work lies in demonstrating that string stability can be effectively achieved using a simple PD control structure and in proposing a design methodology that is straightforward and accessible, enabling practitioners to implement it directly in real-world applications. The proposed method not only contributes to the theoretical understanding of string stability in ACC systems but also serves as a concrete tool for practitioners seeking to implement stable and reliable platoon control using minimal control logic.

### 1.4. Organization of the Paper

The remainder of the paper is organized as follows. Section 2 presents the ACC control systems and formulates the problem. Section 3 derives the conditions for both string stability and individual vehicle stability. The design guideline for the PD controller is developed in Section 4, and the proposed approach is validated in Section 5 through simulation studies. Finally, Section 6 concludes the paper and discusses future research directions, including the extension to heterogeneous vehicles and integration with cooperative control frameworks.

## 2. Problem Formulations

This section formulates the ACC problem addressed in this study. We begin by modeling the longitudinal dynamics of each vehicle and outlining the assumptions under which the control design is developed. Subsequently, we provide formal definitions of key concepts such as individual vehicle stability and string stability. Finally, we describe the desired system behavior and formulate the control objectives accordingly. These formulations serve as the foundation for the controller design developed in the subsequent sections.

### 2.1. Adaptive Cruise Control and Constant Time-Gap Policy

**Figure 1:**
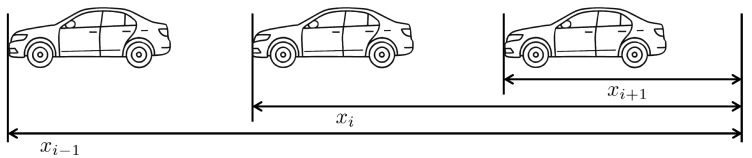
Vehicle following and adaptive cruise control system.

Figure 1 depicts a vehicle following system, where a group of vehicles travels together while maintaining predefined inter-vehicle distances. Each vehicle autonomously adjusts its speed and spacing using on-board sensors, such as radar, LiDAR, or cameras, to ensure safe and efficient following behavior without relying on direct communication with other vehicles. ACC systems serve as a foundational technology for vehicle following, as they enable individual vehicles to autonomously regulate their speed and maintain safe following distances without requiring inter-vehicle communication. By leveraging the on-board sensors, ACC-equipped vehicles can dynamically respond to changes in the behavior of preceding vehicles, thereby supporting the formation and stability of the group of vehicles. In ACC, the CTG spacing policy is commonly employed [34], wherein the desired spacing between vehicles varies linearly with the velocity of the following vehicle. Specifically, the actual spacing between the (i−1)-th and *i*-th vehicles is given by $d_{i}:=x_{i-1}-x_{i}$, ignoring the length of the vehicles, while the desired spacing $d_{d,i}$ is defined as

(1) $$\begin{array}{c} d_{d,i}=hv_{i}:=h{\dot{x}}_{i}, \end{array}$$

where h>0 is the time-gap (also referred to as the headway constant), representing the desired temporal separation between vehicles.

Accordingly, the control objective is to regulate the actual spacing $d_{i}$ to track the desired spacing $d_{d,i}$. This objective can be formulated as minimizing the spacing error $e_{x,i}\left(t\right)$, defined as

(2) $$\begin{array}{c} e_{x,i}\left(t\right):=x_{i-1}\left(t\right)-x_{i}\left(t\right)-h{\dot{x}}_{i}\left(t\right). \end{array}$$

By selecting a suitable time-gap *h*, the desired inter-vehicle distance becomes $h{\dot{x}}_{i}$, and the control goal is to ensure that the spacing error (2) asymptotically converges to zero. The ACC controller will be designed to achieve this spacing objective while preserving stability across the group of vehicles.

### 2.2. Longitudinal Vehicle Dynamics

**Figure 2:**
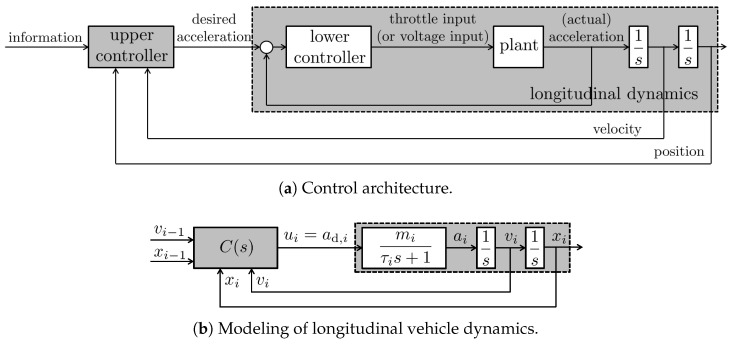
Structure of controller and longitudinal vehicle dynamics.

To achieve effective longitudinal headway distance regulation, a hierarchical two-layer control architecture is typically employed [34], as illustrated in Figure 2a. The upper-level controller computes the desired acceleration based on data collected from sensors. The lower-level controller then translates this desired acceleration into actual actuator commands, such as throttle inputs for internal combustion engines or voltage signals for electric motors [19].

In this study, we focus on the analysis and design of the upper-level controller, which is responsible for generating the desired acceleration command. To this end, the longitudinal vehicle dynamics are modeled to incorporate the servo-loop behavior of the lower-level controller. With the subscript *i* denoting the index of the vehicle in the group, the dynamic relationship from the desired acceleration $a_{d,i}$ to the actual acceleration $a_{i}$ is approximated by a first-order system characterized by a time constant $\tau_{i}$ and a DC gain $m_{i}$. Here, the time constant $\tau_{i}>0$ reflects the response delay associated with driveline dynamics and other mechanical characteristics of the vehicle, while the gain $m_{i}>0$ accounts for variations in curb weight across different vehicles. The resulting model of the longitudinal vehicle dynamics is depicted in Figure 2b.

A fundamental modeling assumption adopted in this work is that all vehicles share identical dynamics, i.e., $m_{i}=m$ and $\tau_{i}=\tau$ for all *i*. Although many studies typically assume m=1, we treat *m* as a parameter that may differ from unity. Accordingly, when the desired acceleration $a_{d,i}$ is regarded as the system input $u_{i}$, and the vehicle position $x_{i}$ is considered as the output $y_{i}$, the resulting longitudinal dynamics can be represented as the following third-order system:

(3) $$\begin{array}{c} P_{i}\left(s\right)=\frac{Y_{i}\left(s\right)}{U_{i}\left(s\right)}=\frac{X_{i}\left(s\right)}{A_{d,i}\left(s\right)}=\frac{1}{s^{2}}\frac{A_{i}\left(s\right)}{A_{d,i}\left(s\right)}=\frac{m}{s^{2}\left(\tau s+1\right)}=:P\left(s\right), \end{array}$$

where capital letters denote the Laplace transforms of the corresponding time-domain variables. Even though the longitudinal model (3) appears to be a simplified representation, it captures the most essential dynamic characteristics of a vehicle’s longitudinal behavior. Consequently, this model has been widely adopted in existing ACC research [34,35,36,37,38,39].

### 2.3. Individual Vehicle Stability and String Stability

For the longitudinal vehicle control in ACC, each vehicle is tasked with maintaining a desired distance from its preceding vehicle. To ensure both safety and efficiency, the control system must satisfy two fundamental performance criteria: individual vehicle stability and string stability [34,40]. Individual vehicle stability refers to the ability of each vehicle to regulate its spacing error, defined as the deviation from the desired inter-vehicle distance, toward zero when the preceding vehicle is traveling at a constant velocity. The formal definition of individual vehicle stability used in this paper is stated as follows.

**Definition 1**  ([40] Section III.B). *A vehicle following system is said to be individually vehicle stable if, for each vehicle i, the spacing error $e_{x,i}\left(t\right)$ in (2) satisfies*

$$\lim_{t\to \infty }e_{x,i}\left(t\right)=0,$$

*provided that the acceleration and control input of the preceding vehicle remain zero, i.e., ${\ddot{x}}_{i-1}\left(t\right)=0$ and $u_{i-1}\left(t\right)=0$ for all t≥0.*

Based on the above definition, individual vehicle stability can be checked by examining the stability of the transfer function from the acceleration of the preceding vehicle, $a_{i-1}:={\ddot{x}}_{i-1}$, to the spacing error $e_{x,i}$, which is given by

(4) $$\begin{array}{c} G_{i}\left(s\right):=\frac{E_{x,i}\left(s\right)}{A_{i-1}\left(s\right)}=\frac{E_{x,i}\left(s\right)}{s^{2}X_{i-1}\left(s\right)}. \end{array}$$

Although individual vehicle stability ensures that each vehicle can regulate its own spacing error, it does not guarantee the overall stability of an ACC-based vehicle following system. In particular, when the preceding vehicle undergoes acceleration changes, tracking errors are inevitable and may propagate downstream. As a result, even if every vehicle is individually stable, the accumulated errors can lead to amplified oscillations or, in extreme cases, collisions. To address this, we consider string stability, which ensures that unexpected changes in driving behavior (such as sudden acceleration or braking maneuvers) do not cause the spacing error to amplify as it propagates from one vehicle to the next in an ACC-based vehicle following system [25,28]. This property is crucial for suppressing traffic oscillations and enhancing the safety and overall traffic flow.

Formally, string stability refers to the condition where the spacing error $e_{x,i}$, defined in (2), does not increase along the vehicle string. Mathematically, string stability is defined by the inequality $∥e_{x,i}∥\leq ∥e_{x,i-1}∥$, which can be expressed as

(5) $$\begin{array}{c} ∥\frac{e_{x,i}}{e_{x,i-1}}∥\leq 1, \end{array}$$

as noted in ([34] Section 6.4). Considering the transfer function that describes the propagation of spacing errors between the (i−1)-th vehicle and the *i*-th vehicle, which is the ratio of the Laplace transforms of $e_{x,i}\left(t\right)$ and $e_{x,i-1}\left(t\right)$,

(6) $$\begin{array}{c} H_{i}\left(s\right):=\frac{E_{x,i}\left(s\right)}{E_{x,i-1}\left(s\right)}, \end{array}$$

applying the condition in (5) to this transfer function $H_{i}\left(s\right)$, leads to the frequency-domain string stability condition as follows.

**Definition 2**  ([28] Section 4.2). *A vehicle following system is said to be string stable if, for each vehicle i, the transfer function of spacing errors between the (i−1)-th vehicle and the i-th vehicle, denoted as $H_{i}\left(s\right)$ in (6), satisfies*

(7) $$\begin{array}{c} {∥H_{i}\left(j\omega \right)∥}_{\infty }\leq 1. \end{array}$$

In this definition, ${∥H_{i}\left(j\omega \right)∥}_{\infty }$ denotes the $H_{\infty }$ norm of $H_{i}\left(j\omega \right)$, that is, the maximum magnitude of the transfer function over all frequencies, and thus, the condition (5) can be written as

$$|H_{i}\left(j\omega \right)|\leq 1,\;\forall \omega \geq 0.$$

If the condition in (7) is satisfied, the vehicle following system is string stable, meaning that spacing errors are not amplified as they propagate along the vehicle string.

### 2.4. Control Problem Statement

We consider a homogeneous group of vehicles governed by the identical first-order longitudinal dynamics augmented with a double integrator, as described in (3). Each vehicle is equipped with on-board sensors, enabling it to measure its own velocity and the relative distance and relative velocity to the preceding vehicle. The desired inter-vehicle spacing is determined by the CTG policy (1), and the control objective is to ensure that the actual spacing $d_{i}\left(t\right)$ accurately tracks the desired spacing $d_{d,i}\left(t\right)=h{\dot{x}}_{i}\left(t\right)$, even under realistic and potentially time-varying acceleration profiles of the lead vehicle. To achieve this, the control input for the *i*-th vehicle can be designed using a PD-type controller, given by

(8) $$\begin{array}{c} u_{i}\left(t\right)=a_{d,i}\left(t\right)=k_{p}\left(x_{i-1}\left(t\right)-x_{i}\left(t\right)-hv_{i}\left(t\right)\right)+k_{d}\left(v_{i-1}\left(t\right)-v_{i}\left(t\right)\right), \end{array}$$

where $k_{p}$ is the proportional gain for relative distance and its own velocity, and $k_{d}$ is the derivative gain for relative velocity.

Under this setting, the vehicle following system must satisfy two key stability criteria to ensure safe and efficient operation:

(1) Individual vehicle stability: Each vehicle must be capable of regulating its own spacing error so that it asymptotically converges to zero when the preceding vehicle travels at a constant velocity, as defined in Definition 1.
(2) String stability: The spacing errors propagated along the vehicle string must not be amplified, ensuring that disturbances introduced by one vehicle do not degrade the performance of downstream vehicles, as formalized in Definition 2.

The objective of this study is to design a PD-based ACC controller that ensures both individual vehicle stability and string stability under the CTG policy while maintaining the predefined inter-vehicle distances. The controller design methodology and theoretical analysis are presented in the following sections.

## 3. Necessary and Sufficient Conditions for Stability

This section begins by analyzing the ACC-based vehicle following system considered in this study and presents the conditions that ensure both individual vehicle stability and string stability. While much research has been conducted on ACC, we revisit existing results and highlight them through the lens of proportional and derivative control gains.

### 3.1. Frequency Domain Analysis of ACC Control System

Assuming that all vehicles share identical longitudinal dynamics, we derive the relevant transfer functions to analyze the stability properties of the control system in the frequency domain. Given that the vehicle dynamics $P_{i}\left(s\right)=P\left(s\right)=\frac{m}{s^{2}\left(\tau s+1\right)}$ described in (3) are homogeneous for all *i*, applying the Laplace transform to the control input designed in (8) yields:

(9) $$\begin{array}{c} \begin{array}{cc} U_{i}\left(s\right) & =k_{p}\left(X_{i-1}\left(s\right)-X_{i}\left(s\right)-hsX_{i}\left(s\right)\right)+k_{d}\left(sX_{i-1}\left(s\right)-sX_{i}\left(s\right)\right)\\ \; & =k_{p}P\left(s\right)\left(U_{i-1}\left(s\right)-U_{i}\left(s\right)-hsU_{i}\left(s\right)\right)+k_{d}sP\left(s\right)\left(U_{i-1}\left(s\right)-U_{i}\left(s\right)\right). \end{array} \end{array}$$

By rearranging (9), we obtain the transfer function from $U_{i-1}\left(s\right)$ to $U_{i}\left(s\right)$, which is independent of the index *i*:

(10) $$\begin{array}{c} \begin{array}{cc} \Gamma \left(s\right):=\frac{U_{i}\left(s\right)}{U_{i-1}\left(s\right)} & =\frac{P\left(s\right)\left(k_{d}s+k_{p}\right)}{1+P\left(s\right)\left(hk_{p}s+k_{d}s+k_{p}\right)}\\ \; & =\frac{m(k_{d}s+k_{p})}{s^{2}\left(\tau s+1\right)+m\left(hk_{p}s+k_{d}s+k_{p}\right)}\\ \; & =\frac{mk_{d}s+mk_{p}}{\tau s^{3}+s^{2}+m\left(hk_{p}+k_{d}\right)s+mk_{p}}. \end{array} \end{array}$$

By utilizing (10), the transfer function $G_{i}\left(s\right)$ in (4), which is used to assess individual vehicle stability, can be derived as follows:

(11) $$\begin{array}{c} \begin{array}{cc} G_{i}\left(s\right): & =\frac{E_{x,i}\left(s\right)}{A_{i-1}\left(s\right)}=\frac{E_{x,i}\left(s\right)}{X_{i-1}\left(s\right)}\frac{1}{s^{2}}\\ \; & =\frac{X_{i-1}\left(s\right)-X_{i}\left(s\right)-hsX_{i}\left(s\right)}{X_{i-1}\left(s\right)}\frac{1}{s^{2}}\\ \; & =\frac{P\left(s\right)\left(U_{i-1}\left(s\right)-U_{i}\left(s\right)-hsU_{i}\left(s\right)\right)}{P\left(s\right)U_{i-1}\left(s\right)}\frac{1}{s^{2}}\\ \; & =\left(1-(hs+1)\Gamma (s)\right)\frac{1}{s^{2}}\\ \; & =\frac{\tau s+\left(1-mhk_{d}\right)}{\tau s^{3}+s^{2}+m\left(hk_{p}+k_{d}\right)s+mk_{p}}=:G\left(s\right). \end{array} \end{array}$$

Notably, it is independent of the vehicle index *i* and is denoted as G(s). To ensure individual vehicle stability, the transfer function G(s) must be stable; that is, its characteristic polynomial must be Hurwitz, meaning all poles lie strictly in the open left half of the complex plane. Accordingly, we define the characteristic polynomial D(s), which is shared by both Γ(s) and G(s), as

(12) $$\begin{array}{c} D\left(s\right):=\tau s^{3}+s^{2}+m\left(hk_{p}+k_{d}\right)s+mk_{p}. \end{array}$$

If D(s) is Hurwitz, then G(s) is stable, and the vehicle following system satisfies individual vehicle stability as defined in Definition 1.

For the analysis of string stability, we derive the transfer function $H_{i}\left(s\right)$ in (6) as follows. Note that it is independent of the vehicle index *i*, and coincides with the transfer function Γ(s) defined in (10).

(13) $$\begin{array}{c} \begin{array}{cc} H_{i}\left(s\right) & :=\frac{E_{x,i}\left(s\right)}{E_{x,i-1}\left(s\right)}=\frac{X_{i-1}\left(s\right)-X_{i}\left(s\right)-hsX_{i}\left(s\right)}{X_{i-2}\left(s\right)-X_{i-1}\left(s\right)-hsX_{i-1}\left(s\right)}\\ \; & =\frac{P\left(s\right)\left(U_{i-1}\left(s\right)-U_{i}\left(s\right)-hsU_{i}\left(s\right)\right)}{P\left(s\right)\left(U_{i-2}\left(s\right)-U_{i-1}\left(s\right)-hsU_{i-1}\left(s\right)\right)}\\ \; & =\frac{\Gamma \left(s\right)\left(U_{i-2}\left(s\right)-U_{i-1}\left(s\right)-hsU_{i-1}\left(s\right)\right)}{U_{i-2}\left(s\right)-U_{i-1}\left(s\right)-hsU_{i-1}\left(s\right)}=\Gamma \left(s\right). \end{array} \end{array}$$

Therefore, the string stability condition (7) leads to the following frequency domain inequality:

(14) $$\begin{array}{c} |\Gamma (j\omega )|\leq 1,\;\forall \omega \geq 0. \end{array}$$

If this condition (14) is satisfied, the vehicle following system is string stable, meaning that spacing errors are not amplified as they propagate downstream through the vehicle string.

### 3.2. Individual Vehicle Stability for ACC

Individual vehicle stability is achieved if and only if the characteristic polynomial D(s) in (12) is Hurwitz. Therefore, the stability condition depends on the selection of $k_{p}$ and $k_{d}$ such that all roots of D(s) lie in the open left half plane of the complex plane. To derive this stability conditions, we apply Routh’s stability criterion ([41] Chapter 3.6) to D(s). According to Routh’s criterion, to ensure the Hurwitz stability of the transfer function, all elements in the first column of the Routh array must have the same sign. The Routh array of D(s), computed as shown in Table 1, leads to the following conditions that must be satisfied to guarantee all elements in the first column remain positive:

(15a) $$\begin{array}{cc} k_{p} & >0, \end{array}$$

(15b) $$\begin{array}{cc} k_{d} & >\left(\tau -h\right)k_{p}. \end{array}$$

By satisfying the conditions in (15), individual vehicle stability is ensured for the vehicle following system.

### 3.3. String Stability for ACC

The string stability condition of the ACC system has been extensively studied in previous research and is well-documented in works such as [27,34]. We revisit these results in this subsection and will provide a refined design guideline for selecting the PD control gains $k_{p}$ and $k_{d}$ in the next section. To ensure string stability, the magnitude of Γ(jω) in (10) must satisfy

(16) $$\begin{array}{c} {\left|\Gamma \left(j\omega \right)\right|}^{2}=\frac{{\left(mk_{p}\right)}^{2}+{\left(mk_{d}\omega \right)}^{2}}{{\left(mk_{p}-\omega^{2}\right)}^{2}+{\left(m\left(hk_{p}+k_{d}\right)\omega -\tau \omega^{3}\right)}^{2}}\leq 1,\;\forall \omega \geq 0, \end{array}$$

as required by the condition (14). This inequality can be rewritten as

$$\begin{array}{cc} \; & \left({\left(mk_{p}-\omega^{2}\right)}^{2}+{\left(m\left(hk_{p}+k_{d}\right)\omega -\tau \omega^{3}\right)}^{2}\right)-\left({\left(mk_{p}\right)}^{2}+{\left(mk_{d}\omega \right)}^{2}\right)\geq 0,\;\;\forall \omega \geq 0,\\ \; & \tau^{2}\omega^{6}+\left(1-2m\tau (hk_{p}+k_{d})\right)\omega^{4}+\left(m^{2}{\left(hk_{p}+k_{d}\right)}^{2}-2mk_{p}-m^{2}k_{d}^{2}\right)\omega^{2}\geq 0,\;\;\forall \omega \geq 0. \end{array}$$

By defining $\chi :=\omega^{2}$, this inequality can be reduced to the following quadratic form:

(17) $$\begin{array}{c} \tau^{2}\chi^{2}+\left(1-2m\tau (hk_{p}+k_{d})\right)\chi +\left(m^{2}{\left(hk_{p}+k_{d}\right)}^{2}-2mk_{p}-m^{2}k_{d}^{2}\right)\geq 0,\;\forall \chi \geq 0. \end{array}$$

With the coefficients

$$\begin{array}{cc} a_{\mathrm{ACC}}: & =\tau^{2}>0,\\ b_{\mathrm{ACC}}: & =1-2m\tau (hk_{p}+k_{d}),\;\mathrm{and}\\ c_{\mathrm{ACC}}: & =m^{2}{\left(hk_{p}+k_{d}\right)}^{2}-2mk_{p}-m^{2}k_{d}^{2}, \end{array}$$

the quadratic function

$$f_{\mathrm{ACC}}\left(\chi \right)=a_{\mathrm{ACC}}\chi^{2}+b_{\mathrm{ACC}}\chi +c_{\mathrm{ACC}}\geq 0\;\mathrm{for}\;\mathrm{all}\;\chi \geq 0,$$

is guaranteed if and only if either of the following conditions is satisfied:

(c1) $b_{\mathrm{ACC}}\geq 0$ and $c_{\mathrm{ACC}}\geq 0$,
(c2) $b_{\mathrm{ACC}}<0$ and $b_{\mathrm{ACC}}^{2}-4a_{\mathrm{ACC}}c_{\mathrm{ACC}}\leq 0$.

First, the condition (c1) can be rewritten as follows:

(18) $$\begin{array}{c} \begin{array}{cccccc} \; & 1-2m\tau (hk_{p}+k_{d})\geq 0 & \; & \mathrm{and} & \; & m^{2}{\left(hk_{p}+k_{d}\right)}^{2}-2mk_{p}-m^{2}k_{d}^{2}\geq 0,\\ \; & hk_{p}+k_{d}\leq \frac{1}{2m\tau } & \; & \mathrm{and} & \; & m^{2}\left(h^{2}k_{p}^{2}+2hk_{p}k_{d}\right)-2mk_{p}\geq 0,\\ \; & hk_{p}+k_{d}\leq \frac{1}{2m\tau } & \; & \mathrm{and} & \; & hk_{p}+2k_{d}\geq \frac{2}{mh}. \end{array} \end{array}$$

By multiplying the second inequality in (18) by $\frac{1}{2}$ and adding $\frac{1}{2}hk_{p}$, this condition (18) can be further simplified to the requirement that the term $hk_{p}+k_{d}$ lies within the following bounds:

(19) $$\begin{array}{c} \frac{1}{mh}+\frac{1}{2}hk_{p}\leq hk_{p}+k_{d}\leq \frac{1}{2m\tau }. \end{array}$$

Second, the condition (c2) can be restated as follows:    

(20) $$\begin{array}{cccccc} \; & 1\;-\;2m\tau (hk_{p}\;+\;k_{d})\;<\;0 & \; & \;\;\;\mathrm{and}\;\;\; & \; & {\left(\;1\;-\;2m\tau (hk_{p}\;+\;k_{d})\;\right)}^{2}\;-\;4\tau^{2}\left(\;m^{2}{\left(hk_{p}\;+\;k_{d}\right)}^{2}\;\;-\;2mk_{p}\;-\;m^{2}k_{d}^{2}\;\right)\;\leq \;0,\;\;\;\;\;\;\\ \; & hk_{p}\;+\;k_{d}>\frac{1}{2m\tau } & \; & \;\;\;\mathrm{and}\;\;\; & \; & {\left(1-2m\tau k_{d}\right)}^{2}-4m\tau k_{p}\left(h-2\tau \right)\leq 0,\\ \; & hk_{p}\;+\;k_{d}>\frac{1}{2m\tau } & \; & \;\;\;\mathrm{and}\;\;\; & \; & {\left(1-2m\tau k_{d}\right)}^{2}\leq 4m\tau k_{p}\left(h-2\tau \right),\\ \; & hk_{p}+k_{d}>\frac{1}{2m\tau } & \; & \;\;\;\mathrm{and}\;\;\; & \; & {\left(k_{d}-\frac{1}{2m\tau }\right)}^{2}\leq \frac{k_{p}}{m\tau }\left(h-2\tau \right). \end{array}$$

Finally, the string stability condition for ACC to satisfy (16) can be summarized as follows:

(21) $$\begin{array}{cccccc} \; & \left(c1\right)\;\left\{\begin{array}{cc} \; & hk_{p}+k_{d}\leq \frac{1}{2m\tau },\\ \; & hk_{p}+k_{d}\geq \frac{1}{mh}+\frac{1}{2}hk_{p}, \end{array}\right.\; & \; & \;\mathrm{or}\;\; & \; & \left(c2\right)\;\left\{\begin{array}{cc} \; & hk_{p}+k_{d}>\frac{1}{2m\tau },\\ \; & {\left(k_{d}-\frac{1}{2m\tau }\right)}^{2}\leq \frac{k_{p}}{m\tau }\left(h-2\tau \right). \end{array}\right. \end{array}$$

**Remark 1.**  *If a conventional ACC system is both individually vehicle stable and string stable, then the time-gap h and the plant time constant τ must satisfy*

(22) $$\begin{array}{c} h\geq 2\tau . \end{array}$$

*In other words, if the time gap h is less than twice the time constant τ, i.e., h<2τ, then the ACC system fails to achieve either individual vehicle stability or string stability. This represents a fundamental limitation of ACC to satisfy the string stability, where the time gap cannot be less than 2τ. The reason for this limitation is explained below. First, under the string stability condition (c1) in (21), it is obtained that*

(23a) $$\begin{array}{cc} \frac{1}{2m\tau } & \geq \frac{1}{mh}+\frac{1}{2}hk_{p}, \end{array}$$

(23b) $$\begin{array}{cc} \frac{1}{2m\tau }-\frac{1}{mh} & \geq \frac{1}{2}hk_{p}>0, \end{array}$$

(23c) $$\begin{array}{cc} \frac{1}{mh\tau }\left(h-2\tau \right) & \geq hk_{p}>0, \end{array}$$

(23d) $$\begin{array}{cc} h-2\tau & >0. \end{array}$$

*Second, if the string stability condition (c2) in (21) holds, it follows that*

(24) $$\begin{array}{c} \begin{array}{cc} \frac{k_{p}}{m\tau }\left(h-2\tau \right) & \geq {\left(k_{d}-\frac{1}{2m\tau }\right)}^{2}\geq 0,\\ h-2\tau & \geq 0. \end{array} \end{array}$$

*It is worth noting that in both derivations, the individual vehicle stability condition (15a) and the basic assumptions h>0, τ>0 are used as prerequisites. Since (23) requires the strict inequality h−2τ>0 and (24) admits h−2τ≥0 as a necessary condition, we deliberately choose h−2τ>0 in both cases to exclude the degenerate boundary scenario and ensure consistency.*

### 3.4. Summary of Stability Conditions

The analytically derived conditions for ensuring individual vehicle stability and string stability are summarized in Table 2. Please note that these parameter conditions will be reformulated into a more accessible form in the next section, where explicit bounds for the control gains will be provided in Equations (36) and (40).

## 4. Design of PD Controller for ACC

The control design problem addressed in Section 2.4 is to determine appropriate control gains $k_{p}$ and $k_{d}$ such that:

(1) The characteristic polynomial D(s) in (12) is Hurwitz, ensuring individual vehicle stability;
(2) The transfer function Γ(s) in (10) satisfies the string stability condition (14).

Based on the stability conditions developed in Section 3, this section presents a systematic methodology for designing the controller that ensures both individual vehicle stability and string stability under the previously established conditions. Based on the PD controller described in (8), we propose a design guideline and systematic procedure for selecting the control gains $k_{p}$ and $k_{d}$ that satisfy both the individual vehicle stability condition in (Section 3.2) and the string stability condition in (21). First, the proportional gain $k_{p}$ is chosen to meet the desired response speed requirements, such as the specified rise time. Next, the derivative gain $k_{d}$ is adjusted to ensure that the stability conditions are satisfied.

### 4.1. Determination of Proportional (P) Gain for ACC

According to the individual vehicle stability condition (15a), the gain $k_{p}$ is greater than zero. As a first step in designing an ACC system, we provide a guideline for selecting the proportional gain $k_{p}>0$ to assist engineers designing an ACC system. This guideline is based on the properties of a standard second-order system.

(25) $$\begin{array}{c} L\left(s\right)=\frac{\omega_{n}^{2}}{s^{2}+2\zeta \omega_{n}s+\omega_{n}^{2}} \end{array}$$

where its properties are very well established [41]. For the standard second-order system L(s) in (25), the rise time $t_{r}$, defined as the time it takes for the system’s response to reach a specified percentage (typically 10% to 90%) of its final value, can be approximately computed as follows:

(26) $$\begin{array}{c} t_{r}\approx \frac{1.8}{\omega_{n}}, \end{array}$$

where $\omega_{n}$ is the undamped natural frequency of the system L(s).

For simplicity, we assume the ideal vehicle dynamics of (3) with τ=0, that is,

(27) $$\begin{array}{c} P_{\mathrm{ideal}}\left(s\right)=\frac{m}{s^{2}}, \end{array}$$

which implies that the actual acceleration instantaneously follows the desired acceleration without delay. With this ideal plant dynamics (27), the transfer function (11) from the acceleration of the preceding vehicle, ${\ddot{x}}_{i-1}$, to the spacing error $e_{x,i}$, becomes

(28) $$\begin{array}{cc} G_{\mathrm{ideal}}\left(s\right)= & =\frac{1-mhk_{d}}{s^{2}+m\left(hk_{p}+k_{d}\right)s+mk_{p}}. \end{array}$$

Noting that $\omega_{n}^{2}=mk_{p}$, by comparing this with (25), we can approximately achieve the desired rise time $t_{d,r}$ that satisfies the requirements of the platoon system by choosing an appropriate $k_{p}$ according to (26) as follows:    

(29) $$\begin{array}{c} \begin{array}{cc} t_{d,r} & \geq t_{r}\approx \frac{1.8}{\sqrt{mk_{p}}},\\ k_{p} & >\frac{1.8^{2}}{m\;t_{d,r}^{2}}. \end{array} \end{array}$$

However, since (29) is based on the idealized assumption τ=0, a certain design margin should be incorporated to account for the model uncertainty.

### 4.2. Determination of Derivative (D) Gain for ACC

Based on the proportional gain $k_{p}$ designed to satisfy (29), it can be expressed as

(30) $$\begin{array}{c} k_{p}=\frac{\lambda }{mh^{2}\tau }\left(h-2\tau \right), \end{array}$$

where the parameter λ>0 is implicitly determined by other system parameters, along with the necessary stability condition h−2τ>0 established in Remark 1.

First, let us consider the case where 0<λ≤1 and denote λ by α in this case. With

(31) $$\begin{array}{c} k_{p}=\frac{\alpha }{mh^{2}\tau }\left(h-2\tau \right),\;\mathrm{where}\;0<\alpha \leq 1, \end{array}$$

the inequality (23c) is satisfied, which corresponds to condition (c1) in (21). Accordingly, the derivative gain $k_{d}$ can be selected to ensure that condition (c1) in (21) is also fulfilled. From the inequality (19), the admissible range of $k_{d}$ can be derived as follows:

(32) $$\begin{array}{c} \begin{array}{cc} \frac{1}{mh}+\frac{1}{2}hk_{p} & \leq hk_{p}+k_{d}\leq \frac{1}{2m\tau },\\ \frac{1}{mh}-\frac{1}{2}hk_{p} & \leq k_{d}\leq \frac{1}{2m\tau }-hk_{p},\\ \frac{1}{mh}-\frac{1}{2}\frac{\alpha }{mh\tau }\left(h-2\tau \right) & \leq k_{d}\leq \frac{1}{2m\tau }-\frac{\alpha }{mh\tau }\left(h-2\tau \right),\\ \frac{1}{mh\tau }\left(\tau -\frac{\alpha }{2}\left(h-2\tau \right)\right) & \leq k_{d}\leq \frac{1}{mh\tau }\left(\frac{h}{2}-\alpha \left(h-2\tau \right)\right). \end{array} \end{array}$$

Since 0<α≤1, it holds that

$$\begin{array}{c} \frac{1}{mh\tau }\left(\frac{h}{2}-\alpha \left(h-2\tau \right)\right)-\frac{1}{mh\tau }\left(\tau -\frac{\alpha }{2}\left(h-2\tau \right)\right)=\frac{1}{mh\tau }\frac{1-\alpha }{2}\left(h-2\tau \right)\geq 0, \end{array}$$

which implies that the inequality (32) is feasible. Therefore, a derivative gain $k_{d}$ satisfying (32) exists and can be appropriately selected. Now, let us derive the condition on $k_{d}$ required to satisfy condition (c2) in (21). Starting from the inequality (20), the bounds on $k_{d}$ can be obtained as follows:

$$\begin{array}{c} \begin{array}{cc} hk_{p}+k_{d}>\frac{1}{2m\tau }\; & \mathrm{and}\;{\left(k_{d}-\frac{1}{2m\tau }\right)}^{2}\leq \frac{k_{p}}{m\tau }\left(h-2\tau \right),\\ k_{d}>\frac{1}{2m\tau }-hk_{p}\; & \mathrm{and}\;\frac{1}{2m\tau }-\sqrt{\frac{k_{p}}{m\tau }\left(h-2\tau \right)}\leq k_{d}\leq \frac{1}{2m\tau }+\sqrt{\frac{k_{p}}{m\tau }\left(h-2\tau \right)},\\ k_{d}>\frac{1}{2m\tau }-\frac{\alpha }{mh\tau }\left(h-2\tau \right)\; & \mathrm{and}\;\frac{1}{2m\tau }-\frac{\sqrt{\alpha }}{mh\tau }\left(h-2\tau \right)\leq k_{d}\leq \frac{1}{2m\tau }+\frac{\sqrt{\alpha }}{mh\tau }\left(h-2\tau \right), \end{array} \end{array}$$

(33) $$\begin{array}{cc} \frac{1}{mh\tau }\left(\frac{h}{2}-\alpha \left(h-2\tau \right)\right) & <k_{d}\leq \frac{1}{mh\tau }\left(\frac{h}{2}+\sqrt{\alpha }\left(h-2\tau \right)\right), \end{array}$$

where the inequality $\alpha \leq \sqrt{\alpha }$, which holds for 0<α≤1, is utilized in the last step. It is trivial to show that the upper bound exceeds the lower bound, which confirms the feasibility of the inequality (33) and guarantees the existence of a $k_{d}$ that satisfies the specified bounds. Since the string stability condition requires that either condition (c1) or (c2) holds, the admissible range of $k_{d}$ is characterized by the union of the bounds provided in (32) and (33) as follows:

(34) $$\begin{array}{cc} \frac{1}{mh\tau }\left(\tau -\frac{\alpha }{2}\left(h-2\tau \right)\right) & \leq k_{d}\leq \frac{1}{mh\tau }\left(\frac{h}{2}+\sqrt{\alpha }\left(h-2\tau \right)\right). \end{array}$$

Moreover, it can be shown that, under this selection, there always exists a value of $k_{d}$ that satisfies the condition (15b) as follows:

(35) $$\begin{array}{c} \begin{array}{cc} h & >0,\\ \frac{h}{2} & >\left(1-\frac{2\tau }{h}\right)\left(-\sqrt{\alpha }h-\alpha \left(h-\tau \right)\right),\\ \frac{h}{2}+\sqrt{\alpha }\left(h-2\tau \right) & >\frac{\alpha }{h}\left(h-2\tau \right)\left(\tau -h\right),\\ \frac{1}{mh\tau }\left(\frac{h}{2}+\sqrt{\alpha }\left(h-2\tau \right)\right) & >\left(\tau -h\right)\frac{\alpha }{mh^{2}\tau }\left(h-2\tau \right),\\ \frac{1}{mh\tau }\left(\frac{h}{2}+\sqrt{\alpha }\left(h-2\tau \right)\right) & >\left(\tau -h\right)k_{p}, \end{array} \end{array}$$

where the second inequality holds because the terms on the right-hand side are negative. Noting that the upper bound of $k_{d}$ in (33) is larger than $\left(\tau -h\right)k_{p}$, one can easily select $k_{d}$ such that $k_{d}>\left(\tau -h\right)k_{p}$. Hence, the final selection of $k_{d}$ for the case in (31), ensuring both individual vehicle stability and string stability, is given by

(36) $$\begin{array}{c} \begin{array}{cc} \max\left\{\frac{1}{mh\tau }\left(\tau -\frac{\alpha }{2}\left(h-2\tau \right)\right),\;\left(\tau -h\right)k_{p}\right\}<k_{d}\; & \leq \frac{1}{mh\tau }\left(\frac{h}{2}+\sqrt{\alpha }\left(h-2\tau \right)\right). \end{array} \end{array}$$

Second, let us consider the case where λ>1, and denote λ by β in this case. With

(37) $$\begin{array}{c} k_{p}=\frac{\beta }{mh^{2}\tau }\left(h-2\tau \right),\;\mathrm{where}\;\beta >1, \end{array}$$

the inequality (23c), corresponding to condition (c1) in (21), cannot be satisfied. Hence, the derivative gain $k_{d}$ should be selected to satisfy condition (c2) in (21). The process described in (33) and (35) can be similarly applied by replacing α with β. In a manner analogous to (33), the following bounds are easily derived:

$$\begin{array}{c} \begin{array}{cc} k_{d}>\frac{1}{2m\tau }-\frac{\beta }{mh\tau }\left(h-2\tau \right)\; & \mathrm{and}\;\frac{1}{2m\tau }-\frac{\sqrt{\beta }}{mh\tau }\left(h-2\tau \right)\leq k_{d}\leq \frac{1}{2m\tau }+\frac{\sqrt{\beta }}{mh\tau }\left(h-2\tau \right), \end{array} \end{array}$$

(38) $$\begin{array}{cc} \frac{1}{mh\tau }\left(\frac{h}{2}-\sqrt{\beta }\left(h-2\tau \right)\right) & \leq k_{d}\leq \frac{1}{mh\tau }\left(\frac{h}{2}+\sqrt{\beta }\left(h-2\tau \right)\right), \end{array}$$

where the inequality $\beta >\sqrt{\beta }$, which holds for β>1, is used, resulting in a different lower bound compared to (33). Furthermore, similar to (35), $k_{d}$ can always be chosen to satisfy the condition (15b) because we have

(39) $$\begin{array}{c} \frac{1}{mh\tau }\left(\frac{h}{2}+\sqrt{\beta }\left(h-2\tau \right)\right)>\left(\tau -h\right)k_{p}. \end{array}$$

Consequently, the final selection of $k_{d}$ for the case in (37), ensuring both individual vehicle stability and string stability, is given by

(40) $$\begin{array}{c} \max\left\{\frac{1}{mh\tau }\left(\frac{h}{2}-\sqrt{\beta }\left(h-2\tau \right)\right),\;\left(\tau -h\right)k_{p}\right\}<k_{d}\leq \frac{1}{mh\tau }\left(\frac{h}{2}+\sqrt{\beta }\left(h-2\tau \right)\right). \end{array}$$

### 4.3. Summary of Design Guideline for ACC

The overall design guideline so far has been summarized in Algorithm 1, and the relevant theoretical foundations and key results supporting this guideline are presented as a theorem in the following.
**Algorithm 1:** Design guideline for individual vehicle stability and string stability**Input:** 
*m*, τ, *h*, $t_{d,r}$**Output:** 
$k_{p}$, $k_{d}$1:Set $k_{p}$ s.t. $k_{p}>\frac{1.8^{2}}{m\;t_{d,r}^{2}}$ (according to (29))2:Let $k_{p}=\frac{\lambda }{mh^{2}\tau }\left(h-2\tau \right)>0$ (according to (30))3:**if **0<λ≤1** then**4:   Set $k_{d}$ s.t. $\max\left\{\;\frac{1}{mh\tau }\left(\tau -\frac{\alpha }{2}\left(h-2\tau \right)\right),\left(\tau -h\right)k_{p}\;\right\}<k_{d}\leq \frac{1}{mh\tau }\left(\frac{h}{2}+\;\sqrt{\alpha }\left(h-2\tau \right)\;\right)$5:   (according to (36))6:**else if **λ>1** then**7:   Set $k_{d}$ s.t. $\max\left\{\;\frac{1}{mh\tau }\left(\;\frac{h}{2}-\;\sqrt{\;\beta }\left(h-2\tau \right)\;\right),\left(\tau -h\right)k_{p}\;\right\}\;<k_{d}\leq \frac{1}{mh\tau }\left(\frac{h}{2}+\;\sqrt{\;\beta }\left(h-2\tau \right)\;\right)$8:   (according to (40))9:**end if**

**Theorem 1.**  *Consider a vehicle following system where each vehicle has identical longitudinal dynamics as described by (3) and is equipped with an ACC controller of the form given in (8). If the control gains $k_{p}$ and $k_{d}$ are selected according to the design guideline outlined in Algorithm 1, then the PD-based ACC vehicle following system under the CTG policy (2) is both string stable and individually vehicle stable.*

*On the contrary, if $k_{p}<0$ or $k_{d}$ is chosen outside the bounds specified in Algorithm 1, the PD-based ACC vehicle following system is neither string stable nor individually vehicle stable.*

**Proof.**  The proof is established through Section 4.1 and Section 4.2. Recall that the gains $k_{p}$ and $k_{d}$ in Algorithm 1 are chosen to satisfy the individual vehicle stability condition (15) and the string stability condition (21). □

## 5. Simulation Results

**Figure 3:**
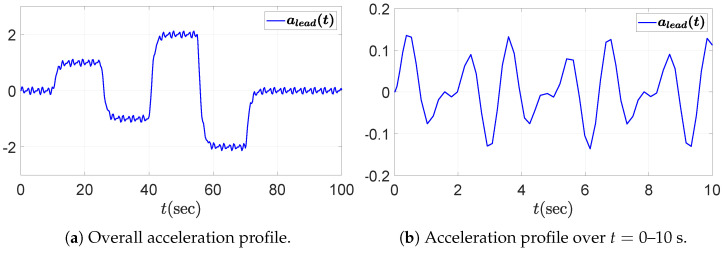
Acceleration profile of the leading vehicle: (**a**) overall view; (**b**) zoomed-in view over the time interval t=0–10 s.

**Figure 4:**
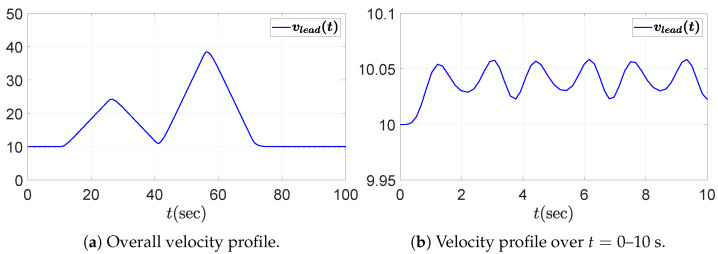
Velocity profile of the leading vehicle: (**a**) overall view; (**b**) zoomed-in view over the time interval t=0–10 s.

We consider a PD-controlled ACC vehicle following system composed of five vehicles, including the leading vehicle. The objective of this simulation is to evaluate the string stability of the vehicle following system by assessing whether disturbances propagate downstream along the vehicle string. The driving scenario involves repeated acceleration and deceleration maneuvers of the leading vehicle, accompanied by fluctuations due to external disturbances. This setting is deliberately chosen because fluctuations in the leader’s acceleration can induce spacing or velocity errors that may amplify toward the rear of the string if the system is not string stable. Accordingly, we assess whether the following four vehicles maintain string stability under such dynamic conditions. The acceleration and velocity profiles of the leading vehicle are presented in Figure 3 and Figure 4, respectively, including both the overall view and a zoomed-in view over the time interval t=0 to 10 s. The fluctuation in the leading vehicle’s acceleration, depicted in the zoomed-in view of Figure 3b, is given by the sum of sinusoidal functions.

(41) $$\begin{array}{c} a_{fluc}\left(t\right)=0.1\left(\sin\left(4t\right)+\sin\left(6t\right)\right). \end{array}$$

To ensure string stability of the ACC-based vehicle following system, the control gains are selected according to the design guideline presented in Algorithm 1. The plant DC gain *m*, the plant time constant τ, and the design requirements on the time-gap *h* are specified as follows

(42) $$\begin{array}{c} m=1,\;\tau =0.2,\;h=0.5. \end{array}$$

Assuming a vehicle string composed of cars characterized by the parameters in (42) and governed by the longitudinal dynamics in (3), we design a PD controller in the form of (8) and conduct simulations for various combinations of the control gains $k_{p}$ and $k_{d}$. For the first case study, we set the desired rise time to $t_{d,r}=3$ s. According to (29), the proportional gain $k_{p}$ must satisfy

$$k_{p}>0.36.$$

We choose $k_{p}=0.8$, and (30) then yields λ=0.4, which is less than unity. Consequently, the allowable range for the derivative gain $k_{d}$ determined by (36) is

(43) $$\begin{array}{c} 1.8<k_{d}\leq 3.1325. \end{array}$$

**Figure 5:**
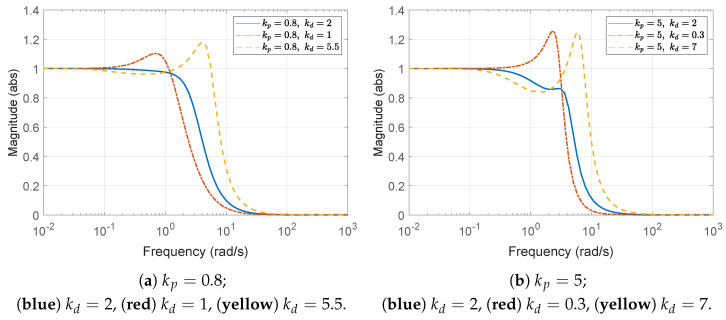
Bode magnitude plot of Γ(jω).

**Figure 6:**
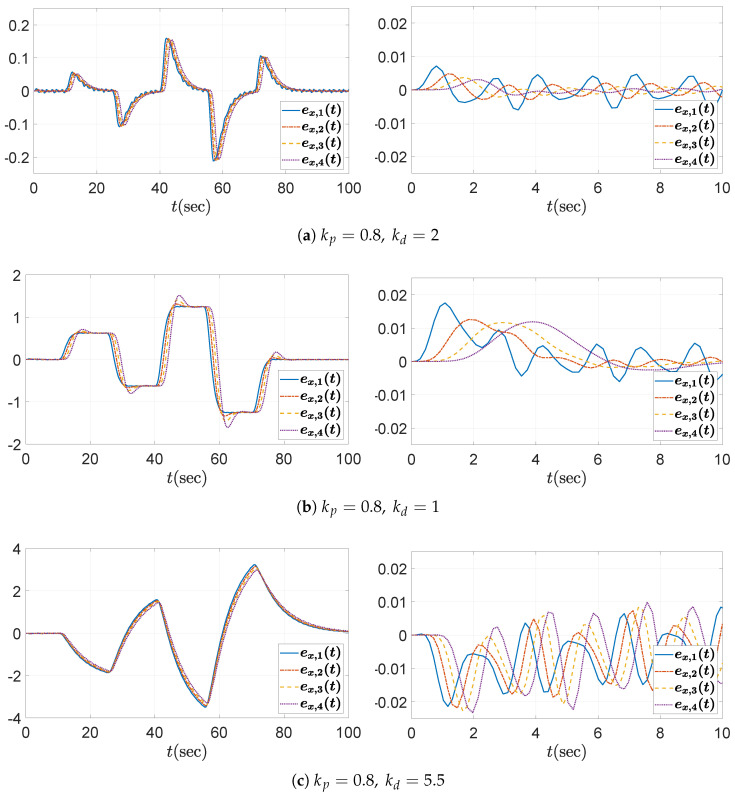
Headway distance error $e_{x,i}\left(t\right)$ with $k_{p}=0.8$: (**left**) overall view; (**right**) zoomed-in view over the time interval t=0–10 s.

Three values for the derivative gain are considered: (1) $k_{d}=2$, (2) $k_{d}=1$, and (3) $k_{d}=5.5$. As anticipated from the above bounds (43), $k_{d}=2$ satisfies the string stability condition, whereas $k_{d}=1$ and $k_{d}=5.5$ do not. This can be initially verified from the Bode plot of the transfer function Γ(s) defined in (10), as shown in Figure 5a. It can be observed that the condition ${∥\Gamma \left(j\omega \right)∥}_{\infty }\leq 1$ is satisfied only when $k_{d}=2$. In addition, the time histories of the spacing errors are shown in Figure 6, with the corresponding zoomed-in views over the interval t=0 to 10 s presented in the right panels. It is evident that the spacing error decreases toward the rear of the vehicle string only in the case where $k_{d}=2$. Moreover, during periods of constant acceleration (e.g., over the intervals t=15–25 s and t=30–40 s, excluding the small fluctuations introduced by (41)), the steady-state spacing error converges to zero only when $k_{d}=2$. This behavior can be explained by the fact that the DC gain of the transfer function G(s) in (11) becomes $\frac{1-mhk_{d}}{mk_{p}}=0$ for the chosen value $k_{d}=2$. Although the *y*-axis scale in each subplot has been adjusted to improve visual clarity, it is still evident that the control parameters selected according to the proposed guideline effectively reduce the amplitude of the spacing errors. For the large derivative gain $k_{d}=5.5$, whose corresponding plots are presented in Figure 6c, it is also worth noting that the spacing errors $e_{x,i}\left(t\right)$ may appear to satisfy string stability when viewed on the overall scale (left panel), despite the fact that this case does not meet the string stability condition. However, a closer examination reveals otherwise. The input signal, shown in Figure 3b and defined in (41), contains a dominant frequency component near 4rad/s, which corresponds to the peak with gain greater than unity in the Bode plot in Figure 5a. As a result, the corresponding spacing errors are amplified along the vehicle string, as illustrated in the zoomed-in view (right panel) of Figure 6c.

For the second case study, we set the desired rise time to $t_{d,r}=0.9$ s. Using (29), the proportional gain $k_{p}$ must satisfy the inequality

$$k_{p}>4.$$

We select $k_{p}=5$, which leads to λ=2.5 as determined by (30), a value greater than unity. As a result, the permissible range for the derivative gain $k_{d}$ is given by (40) as

(44) $$\begin{array}{c} 0.919<k_{d}\leq 4.081. \end{array}$$

**Figure 7:**
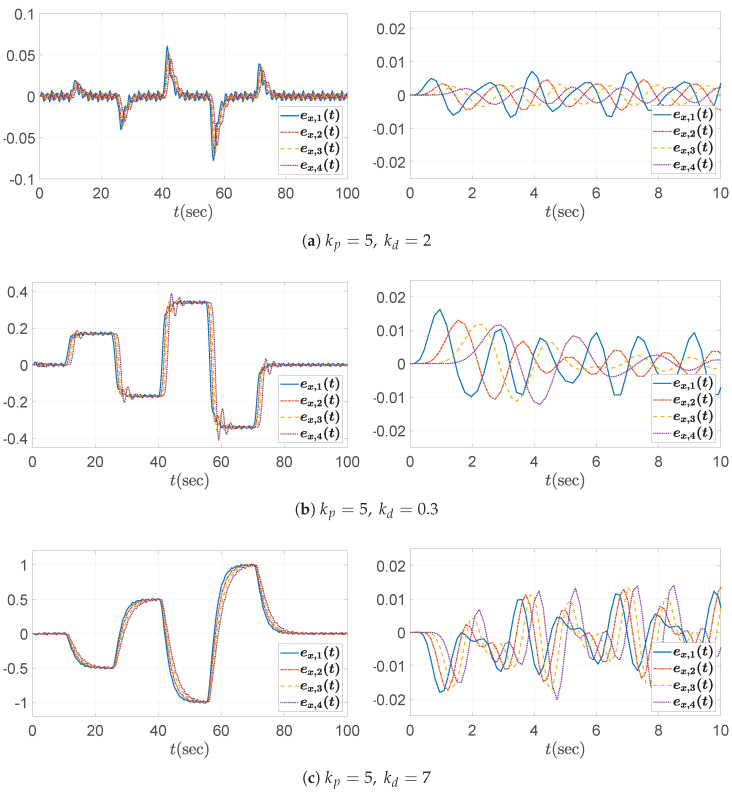
Headway distance error $e_{x,i}\left(t\right)$ with $k_{p}=5$: (**left**) overall view; (**right**) zoomed-in view over the time interval t=0–10 s.

Three specific values for the derivative gain are chosen: (1) $k_{d}=2$, (2) $k_{d}=0.3$, and (3) $k_{d}=7$. As expected from the bounds in (44), $k_{d}=2$ satisfies the string stability condition, whereas both $k_{d}=0.3$ and $k_{d}=7$ do not. This is initially confirmed by examining the Bode plot of the transfer function Γ(s) in Figure 5b. From the plot, it is clear that the condition ${∥\Gamma \left(j\omega \right)∥}_{\infty }\leq 1$ is only satisfied when $k_{d}=2$. Furthermore, Figure 7 shows the time histories of the spacing errors, with their corresponding zoomed-in views over the initial interval t=0 to 10 s presented in the right panels. Among the tested cases, only the configuration with $k_{d}=2$ consistently exhibits a reduction in spacing error along the vehicle string. Similar to the previous case, during periods of constant acceleration (e.g., over the intervals t=15–25 s and t=30–40 s, excluding the small fluctuations introduced by (41)), the steady-state spacing error converges to zero only when $k_{d}=2$. This outcome is consistent with the DC gain of the transfer function G(s) in (11), which evaluates to $\frac{1-mhk_{d}}{mk_{p}}=0$ for this particular gain selection of $k_{d}=2$. Although the *y*-axis scales differ across subplots to enhance visual clarity, it is evident that the control gains chosen according to the proposed guideline successfully reduce the amplitude of the spacing errors. In contrast, for the large derivative gain case of $k_{d}=7$, the error profiles may initially appear to satisfy the string stability condition from the overall scaled view (left panel) in Figure 7c. However, closer inspection reveals that this is not the case. Specifically, the fluctuation input signal (see (41) and Figure 3b) contains a significant spectral component near 6rad/s, which coincides with a peak exceeding unity in the Bode plot shown in Figure 5b. As a result, these frequency components are amplified as they propagate along the vehicle string, as clearly illustrated in the zoomed-in view (right panel) of Figure 7c.

We have summarized all the tested combinations of control gains and evaluated their corresponding stability conditions in Table 3, which serves as the basis for the simulation studies. Note that individual vehicle stability requires both conditions in (15a) and (15b) to be satisfied, whereas string stability is ensured if either condition (c1) or (c2) in (21) holds. Specifically, among the string stable cases, the control gain set $k_{p}=0.8$, $k_{d}=2$ satisfies condition (c1) in (21) (equivalently, (32), which leads to (36)), whereas the control gain set $k_{p}=5$, $k_{d}=2$ satisfies condition (c2) in (21) (equivalently, (38), which leads to (40)).

## 6. Conclusions

In this paper, we propose a systematic approach to control gain selection in ACC systems, ensuring both individual vehicle stability and string stability for a vehicle following system with identical longitudinal dynamics. The control architecture considered here is based on a basic PD controller, where the proportional gain $k_{p}$ and derivative gain $k_{d}$ must be carefully selected to satisfy the stability criteria. The primary contribution of this work lies in establishing the string stability of ACC systems using a fundamental PD control structure under the CTG policy, which is widely adopted for its simplicity and ease of implementation in practical applications. We begin by revisiting and rigorously analyzing the necessary and sufficient conditions for selecting $k_{p}$ and $k_{d}$ that guarantee both individual vehicle stability and string stability. Building on this analysis, we provide a comprehensive and practical design guideline for control gain selection. This framework offers a valuable tool for practitioners in the field of ACC, facilitating the selection of appropriate control parameters for real-world platooning scenarios. In doing so, this paper contributes to both the theoretical understanding of string stability and the practical implementation of string-stable ACC systems.

Nevertheless, this study proposes an analytical controller design method under the assumption of a homogeneous vehicle platoon with a simplified longitudinal model. Further research is required to evaluate the impact of this assumption in real-world implementations. In particular, incorporating more realistic dynamics, model uncertainties, and system heterogeneity—such as unmodeled dynamics, sensor delays, and inter-vehicle variability—could enhance the practical relevance of the proposed approach and underscore the need for novel control design methodologies that can address such complexities and improve robustness. Therefore, future work should focus on extending the applicability of the proposed parameter selection framework to uncertain heterogeneous platoons by developing robust control strategies. Techniques such as disturbance observers (DOB) can be employed to mitigate model uncertainties and external disturbances, enabling the system to behave as if it were a nominal disturbance-free model [42,43,44,45]. Additionally, future research could build upon this work by incorporating V2V communication into the proposed framework, particularly within the context of CACC. Integrating feedforward control based on shared information via V2V communication has the potential to further improve string stability and robustness, thereby enabling shorter headway distances between vehicles in a platoon. To fully leverage these benefits, a rigorous analysis and systematic design methodology for CACC with feedforward control are required.

## Figures and Tables

**Table 1:** Routh array of D(s).

$s^{3}\;$	τ	$\;m(hk_{p}+k_{d})$
$s^{2}\;$	1	$\;mk_{p}$
$s^{1}\;$	$\;m\left(hk_{p}+k_{d}\right)-m\tau k_{p}\;$	0
$s^{0}\;$	$\;mk_{p}$	

**Table 2:** Conditions for individual vehicle stability and string stability.

Class	Transfer Function	Original Condition	Condition on Parameter
Individual stability	G(s) in (11)	(12) is Hurwitz	(15)
String stability	Γ(s) in (10)	(14)	(21) (equivalently, (36) or (40))

**Table 3:** Feasibility check of individual vehicle stability and string stability under given parameters.

Model Parameters			Control Gains		Individual Stability		String Stability	
m	τ	h	$k_{p}$	$k_{d}$	(15a)	>(15b)	(c1) in (21)	(c2) in (21)
1	0.2	0.5	0.8	2	◯	◯	◯	×
				1	◯	◯	×	×
				5.5	◯	◯	×	×
			5	2	◯	◯	×	◯
				0.3	◯	◯	×	×
				7	◯	◯	×	×

## Data Availability

Data are contained within the article.
